# Functional divergence of MdpS and MdpS2 reveals mucin-targeting strategies in *Streptococcus oralis*

**DOI:** 10.1080/20002297.2025.2571186

**Published:** 2025-10-26

**Authors:** Fredrik Leo, Jonas Nilsson, Liisa Arike, Sahana Kumar, Emma Hilton, Rolf Lood, David J. Thornton, Gregg A. Duncan, Gunnel Svensäter, Claes Wickström

**Affiliations:** a Department of Oral Biology and Pathology, Faculty of Odontology, Malmö University, Malmö, Sweden; b Genovis AB, Kävlinge, Sweden; c Proteomics Core Facility, Sahlgrenska Academy at the University of Gothenburg, Gothenburg, Sweden; d Fischell Department of Bioengineering, University of Maryland, College Park, MD, United States; e Manchester Cell Matrix Centre and Lydia Becker Institute of Immunology and Inflammation, School of Biological Sciences, Faculty of Biology, Medicine and Health, Manchester Academic Health Sciences Centre, University of Manchester, Manchester, United Kingdom; f Department of Clinical Sciences Lund, Division of Infection Medicine, Faculty of Medicine, Lund University, Lund, Sweden

**Keywords:** *Streptococcus oralis*, mucin degradation, MUC5B, biofilm dispersal, microbial adaptation, protease, mucus rheology, oral microbiome, MdpS, MdpS2

## Abstract

**Background:**

Mucin degradation is essential for understanding oral microbial adaptation, yet the enzymes involved remain incompletely understood. Herein, we have characterised two mucin-degrading proteases, MdpS and MdpS2, from the oral commensal *Streptococcus oralis.*

**Materials and methods:**

MdpS2 was characterised using physicochemical assays and substrate profiling and was compared to MdpS. Further Mdp characterisation included structural modelling, and functional assays analysing the gene expression during biofilm growth on salivary MUC5B, enzyme-induced biofilm dispersal, and mucus degradation analysed through nanoLC-MS/MS, sedimentation profiling, and microrheology.

**Results:**

MdpS2 shared conformational homology with MdpS despite low sequence identity and showed greater tolerance to pH and sodium chloride. Both genes were significantly upregulated during late stationary biofilm phase. MdpS and MdpS2 hydrolysed MUC5B extensively, with overlapping but distinct hydrolysis patterns. MdpS2 promoted biofilm dispersal and caused a pronounced reduction in MUC5B size and compactness. Microrheology showed selective modulation of MUC5B-rich mucus by MdpS2, while MdpS affected both MUC5B and MUC5AC networks.

**Conclusions:**

MdpS and MdpS2 exhibit complementary biochemical and functional profiles, supporting their roles in mucin degradation and biofilm remodelling. These findings advance our understanding of how early colonizing streptococci may interact with mucosal surfaces, influence biofilm dynamics and oral ecology, and suggest potential applications in targeting mucus-related disorders.

## Key messages

• MdpS and MdpS2 are functionally complementary proteases that enable *Streptococcus oralis* to degrade salivary mucins.
• MdpS2 selectively modulates MUC5B-rich mucus, disrupts MUC5B networks, and can promote biofilm dispersal.
• These findings suggest that MdpS and MdpS2 may contribute to mucosal adaptation and biofilm remodelling, with potential applications in managing mucus-related disorders.

## Introduction

The human oral cavity harbours a diverse microbial community, with streptococci playing a pivotal role in shaping the composition and dynamics of the oral microbiome. Among these, species belonging to the *Streptococcus mitis* Group (SMG), including *S. mitis*, *S. oralis*, and *S. sanguinis*, are abundant early colonisers that contribute to biofilm formation and oral homeostasis [1,2]. These commensal streptococci establish multispecies biofilms on tooth and mucosal surfaces where they engage in complex interactions with the host and other microbial species [1]. Their ability to adhere to salivary pellicles, aggregate with neighbouring bacteria, and modulate immune responses plays a crucial role in maintaining a balanced oral environment [3]. Although there is growing evidence suggesting an opportunistic role of *S. oralis* in infectious diseases [4], it is primarily regarded as a commensal species essential for oral health. *S. oralis* contributes to the formation and stability of oral biofilms, which support a balanced ecosystem, and help prevent colonisation by pathogenic species [5]. However, under dysbiotic conditions, members of SMG can act as opportunistic pathogens, facilitating the transition from a healthy biofilm to a disease-associated state [4,6]. The interplay between SMG species, host factors, and microbial competitors is key to understanding the mechanisms that regulate the oral microbial ecology [6]. Elucidating these processes provides valuable insights into the maintenance of oral health and pathogenesis of streptococcal infections.

MUC5B, the predominant gel-forming mucin in saliva, plays a crucial role in protecting mucosal surfaces by trapping pathogens and particular matter, thereby serving as a key barrier in the oral cavity [7]. It contributes to the viscoelastic properties of saliva, enabling lubrication of oral surfaces while preventing pathogen colonisation and infection by downregulating quorum-sensing pathways and inhibiting biofilm formation [8–11]. The glycosylation pattern of MUC5B is vital for its function, as modifications can influence its protective properties [9]. Additionally, its O-glycans serve as nutrient sources for commensal bacteria, supporting a balanced oral microbiome [12]. Beyond the oral cavity, MUC5B is also prevalent in other mucosal tissues, including the respiratory tract, where it functions alongside MUC5AC as a part of the mucus gel [13]. The structural organisation of these mucins determines the viscoelastic properties of airway mucus, and the ratio between them is tightly regulated to ensure effective mucociliary clearance [13]. Although further research is needed, evidence suggests that MUC5B primarily forms long bundle-like strands, whereas MUC5AC appears as thin threads or sheets, typically upregulated during infections [14].

In the oral cavity, MUC5B plays a pivotal role in the formation of the salivary enamel pellicle through protein-protein interactions with the initial pellicle components and larger proteins, such as sIgA and *α*-amylase, forming a hydrophilic layer [15,16]. Within the salivary pellicle, MUC5B forms an intricate network where its protein backbone and extensive glycan structures provide a scaffold for microbial adhesion [16,17]. While this network is essential for maintaining the structural integrity of salivary mucus, it also presents an opportunity for bacterial colonisation, particularly by early colonising species such as *S. oralis*, which can adhere to and initiate biofilm formation [17,18]. Biofilm formation is initiated when *S. oralis* and other early colonising streptococci adhere to MUC5B in the pellicle, a process mediated by sortase A, which anchors surface proteins to the peptidoglycan layer of the bacterial cell wall [19]. Among these, the sialic acid-binding protein AsaA plays a key role in recognising and binding sialylated structures on MUC5B [20]. In addition to surface proteins, A-type sortases also anchor pili, such as the PI-2 pilus, to the cell wall which further mediates adhesion to the salivary pellicle [21]. The PI-2 pilus consists of the tip adhesin PitA and the backbone pilin PitB and is polymerised by C-type sortases SrtG1 and SrtG2 [21]. As the biofilm matures and microbial diversity increases, the protein backbone and extensive glycan structures of MUC5B may serve as nutrient sources, facilitated by the coordinated activity of glycosidases and proteases from various bacterial species [22]. However, the extent to which this degradation influences the network-forming capacity of MUC5B and its role in biofilm structural dynamics remains unclear.

Although mucin degradation by intestinal bacteria has been extensively studied, the enzymes responsible for MUC5B degradation are poorly understood. The discovery of MUC5B-degrading proteases MdpL from *Limosilactobacillus fermentum* [23] and MdpS from *S. oralis* [24] introduced a new class of mucin-targeting proteases distinct from mucinases, such as StcE, TagA, Pic, and SmE [25–28]. Unlike mucinases, MdpL and MdpS hydrolyse mucin-like substrates independently of O-glycans with no strict amino acid preference [23,24]. Notably, MdpS is highly conserved among oral streptococcal species, suggesting its specialised role in bacterial adaptation and biofilm survival. Although the physicochemical properties of these Mdp proteases responsible for extensive MUC5B hydrolysis have been described, their specific biological impact remains unclear, necessitating further investigation to fully understand their broader biological functions.

In this study, we investigate the functional roles of two mucin-degrading proteases, MdpS and the newly identified MdpS2, putatively encoded in the same operon in *Streptococcus oralis*. While MdpS has been partially characterised, its biological relevance and functional properties remain incompletely understood. To address this, we employed a multi-level experimental approach combining structural modelling, physicochemical assays, biofilm growth and gene expression analysis, enzyme-induced biofilm dispersal assays, profiling of MUC5B degradation, sedimentation-based MUC5B size distribution analysis, and microrheological measurements. These complementary methods enabled us to characterise previously unexplored structural, biochemical, and functional aspects of both MdpS and MdpS2.

## Material and methods

### Genomic analysis of the *mdp* operon

The genome of *S. oralis* American Type Culture Collection (ATCC) 9811 (accession number: assembly_19170766374741d0_1) was analysed in MacVector (v18.5.1) (MacVector Inc., USA) to study the ‘*mdp* operon’ and its neighbouring genes to understand the genomic context of the previously characterised MdpS enzyme [24]. The reverse complement sequence was analysed with BPROM (Softberry Inc., United States) to identify potential sigma70 promotor regions. BPROM uses a linear discriminant function to accurate promotor prediction. To increase the confidence in these predictions, a cross-validation using iPromoter-2L was performed [29]. This machine-learning-based algorithm applies multi-window pseudo k-tuple nucleotide composition and Random Forest classification for sigma promoter detection. ARNold (v. 2.0) was used to predict Rho-independent transcription terminators, employing Erpin and RNAmotif algorithms for precise detection [30]. Predicted amino acid sequences of the *mdp* operon and neighbouring genes were queried using Basic Local Alignment Search Tool for Proteins (BLASTP) against the NCBI non-redundant protein database to identify homologous proteins for potential recombinant expression in *Escherichia coli*. To assess the conservation of the *mdp* operon across related *Streptococcus* species, the genomic context was analysed using the BioCyc Genome Browser (v. 29.0, SRI International, United States) [31]. Orthologous regions were compared across selected genomes, with *S. oralis* ATCC 35037 used as the reference due to the unavailability of the ATCC 9811 genome in the BioCyc database. The gene *mdpS2,* annotated *in silico* as *tsaB,* was used as the reference point for comparative analysis. Annotations based solely on *in silico* predictions should be interpreted with caution and no functional claims should be made, particularly if a low overall sequence identity to characterised proteins is identified. Gene annotations and operon structures were visually inspected to identify conserved synteny and potential *mdp* operon alterations in other species.

### Recombinant expression and purification of MdpS and MdpS2

Codon-optimised gene constructs for the whole MdpS (GenBank accession number: WP_084852800.1) and MdpS2 (WP_139689688.1 respectively) without predicted signal peptide were inserted into a pET21 (a) plasmids (Genscript, United States). The FASTA sequences can be found in Supplementary Figures 1 and 2. Expression and purification were performed as previously described [24]. Briefly, constructs were recombinantly expressed in *E. coli* BL21 (DE3) STAR cells as fusion proteins with a C-terminal Gly-Ser-Gly (GSG) linker and 6xHis-tag. Bacteria were cultured in Luria Broth at 37 °C until the optical density at 600 nm (OD_600_) reached 0.6, then induced with 1 mM isopropyl *β*-D−1-thiogalactopyranoside (IPTG) for 5 hours. Cells were collected, frozen, thawed, and then lysed in His binding buffer (20 mM NaP, 500 mM NaCl, 20 mM imidazole, pH 7.4) using sonication. After centrifugation, the supernatant was purified using GraviTrap columns (Cytiva, United States) and eluted with His elution buffer (20 mM NaP, 500 mM NaCl, 500 mM imidazole, pH 7.4). The proteins were re-buffered to Tris Buffered Saline (TBS), and their concentration and purity were determined using a Nanodrop spectrophotometer (Thermo Fisher Scientific, United States) and Sodium Dodecyl Sulphate Polyacrylamide Gel Electrophoresis (SDS-PAGE). Aliquots of 30 μg were stored at −20 °C to prevent aggregation and activity loss.

**Figure 1.**
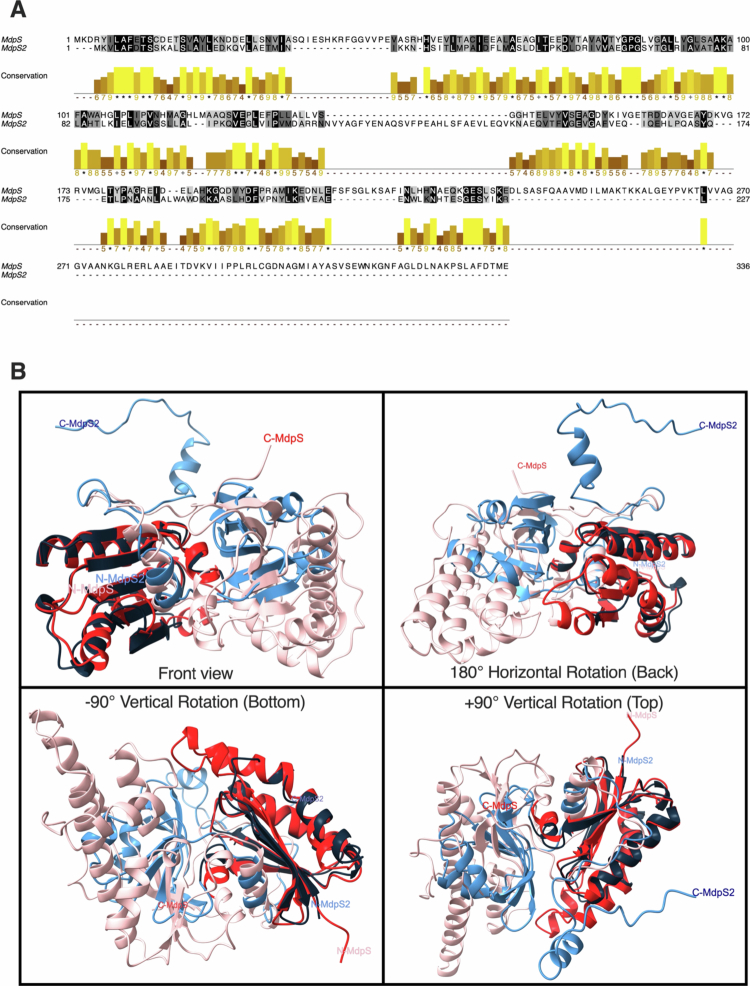
Primary and tertiary sequence alignment of MdpS and MdpS2. (A) Multiple sequence alignment of MdpS and MdpS2 generated using Clustal Omega and visualised in Jalview. Gaps are indicated by dashes (−). Level of sequence identity is indicated by a white-to-black gradient represents sequence identity, with black indicating identical residues. Conservation scores are shown as yellow bars, where height corresponds to the degree of similarity between aligned residues according to clustal scores: low (score 4−6), moderate (7−8), high (9), very high ( + ), and full conservation (*). (B) Predicted tertiary structures of MdpS (pink) and MdpS2 (light blue) aligned using ChimeraX. Structurally similar *N*-terminal domains are highlighted in darker colours: red for MdpS (residues 1−119) and dark blue for MdpS2 (residues 1−100). Structures were predicted using AlphaFold and visualised using ChimeraX.

**Figure 2.**
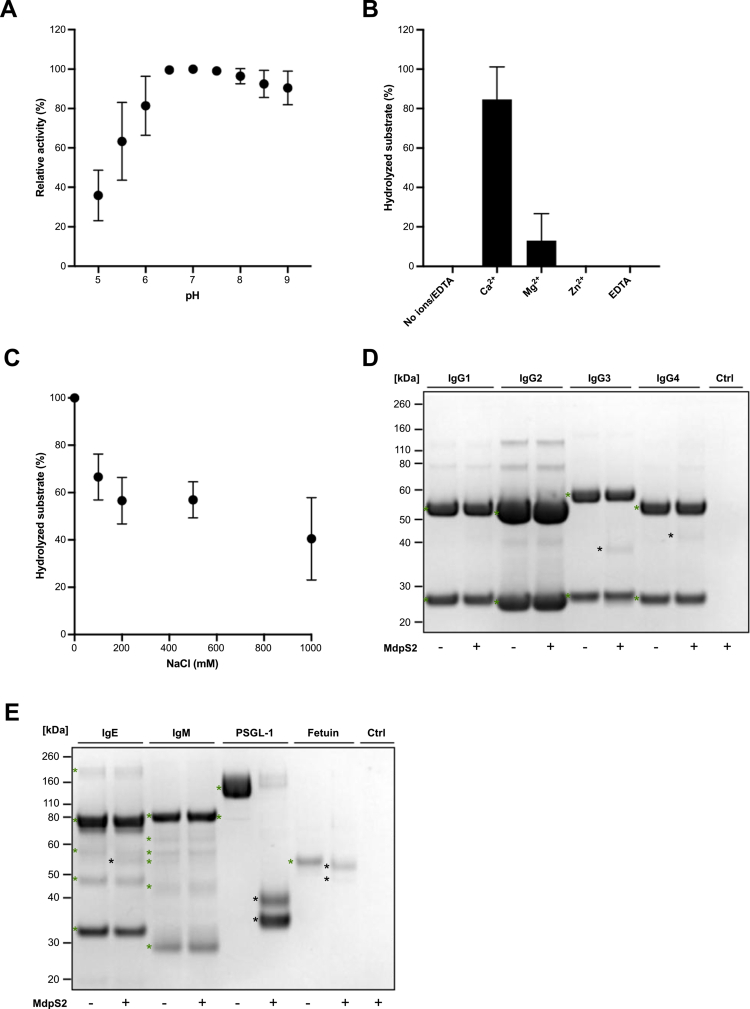
Impact of various physicochemical conditions on MdpS2 and its substrate specificity. The effects were analysed with SDS-PAGE under different conditions: (A) across a pH range (5.0 to 9.0), (B) in the presence of 2 mM Ca^2+^, Mg^2+^, Zn^2+^, or EDTA, (C) under increasing sodium chloride concentrations (0−1000 mM). All assays were performed in technical triplicates using SDS-PAGE and densitometric quantification. The percentage of relative activity in panel A represents the calculated amount of hydrolysed substrate compared to the highest value in the data set. The percentage of hydrolysed substrate in panel B and C represents the calculated amount of digested substrate. Baseline controls for panel A showed 96.0% substrate hydrolysis. Data in panels A-C are presented as mean values ± standard deviation (SD). MdpS2 substrate specificity was assessed by measuring activity towards (D) human IgG subclasses, and (E) human IgE, IgM, and O-glycoproteins. All substrates were incubated (18 h, 37 °C) with pre-activated MdpS2 and were analysed using SDS-PAGE under reducing conditions. Intact substrate and MdpS2-generated fragments are highlighted with a green and black asterisk, respectively.

### Comparing the primary and tertiary structures of MdpS and MdpS2 using Jalview, AlphaFold and ChimeraX

Pairwise sequence alignment was performed using Jalview (v.2.11.4.1) [32] with the Clustal Omega algorithm (v.1.2.4). The protein sequences of MdpS (Genbank: WP_084852800.1) and MdpS2 (Genbank: WP_139689688.1) were aligned, introducing gaps to optimise the alignment. Identical residues, and semi-conserved substitutions were annotated with a white-to-black colour gradient, and conservation scores (4−9, + , *) were highlighted with yellow bars.

The three-dimensional structures of MdpS and MdpS2 were predicted using AlphaFold (v. 3.0.1, DeepMind) with default parameters [33,34]. Protein sequences in FASTA format were used as input, and the model utilised pre-trained weights and multiple sequence alignments from Uniprot, MGnify, and BFD databases. Monomer and multimer models were generated and evaluated using pIDDT scores and PAE plots. The 3D coordinates in PDB format were visualised and analysed using ChimeraX (v. 1.9, UCSF). Structural alignment was performed with the matchmaker command, assigning distinct colours to each model (MdpS in white, MdpS2 in black) and highlighting a similar domain in light and dark green. The tertiary structural similarity of the entire sequences, the highlighted domain, and the non-similar region was quantified by calculating the root-mean-square deviation (RMSD) in ChimeraX.

### Physicochemical characterisation of MdpS2

A similar characterisation approach to what has been previously described for MdpS [24] was carried out. The effect of cations/ethylenediaminetetraacetic acid (EDTA) (2 mM CaCl_2_, MgCl_2_, ZnCl_2_, EDTA) on non-preactivated MdpS2 activity toward etanercept (Pfizer Inc., United States) in 50 mM Tris (pH 7.0) was determined using SDS-PAGE with densitometric quantification using Image Lab software (v. 6.0, Bio-Rad laboratories, United States). The effect of NaCl (0−1000 mM) was assessed using pre-activated MdpS2, incubated (24 h, 37 °C) in an activity buffer (2 mM CaCl_2_, 50 mM Tris (pH 7.0)) prior to NaCl addition. The pH optimum was determined using pre-activated MdpS2 in 100 mM sodium acetate buffer (pH 5.0−5.5), 50 mM Bis-Tris (pH 6.0−6.5), or 50 mM Tris (pH 7.0−9.0), followed by densitometric quantification. The amount of hydrolysed etanercept was calculated by dividing the separate measured activity values by the unhydrolyzed substrate control. In all assays, MdpS2 was incubated with etanercept (18 h, 37 °C) in technical triplicates.

### Substrate specificity of MdpS2

The substrate-specific activity was measured with densitometric quantification from reduced SDS-PAGE, after pre-activated MdpS2 incubation with IgG1−4, IgE, IgM, PSGL−1, and fetuin in 50 mM Tris, pH 7.0, with 2 mM Ca^2+^ (18 h, 37 °C). Humanised IgG1 trastuzumab (Roche, Switzerland), human IgG2 panitumumab (Amgen, United States), human plasma IgG3 myeloma (Merck, Germany), human IgG4 nivolumab (Bristol-Myers Squibb, United States), human IgE myeloma (Merck), human serum IgM (Merck), recombinant human PSGL−1/CD162 Fc chimera protein (Bio-Techne, Ireland), foetal calf serum fetuin (NEB, United States), were used.

### Comparing the rate of hydrolysis between MdpS and MdpS2

To assess the self-activation and substrate hydrolysis rate of MdpS and MdpS2, both enzymes were incubated with etanercept in an activity buffer (2 mM CaCl_2_, 50 mM Tris (pH 7.0)) at a 1:1 enzyme-to-substrate ratio (w/w). Incubations were conducted at 37 °C and sampled at 2, 6, 16, and 24-hour intervals. Enzymatic activity was evaluated by comparing the amount of unhydrolyzed substrate between MdpS and MdpS2 at each time point.

### Bacterial strain

The strain *Streptococcus oralis* ATCC 9811, used in this study, was purchased from ATCC (United States). The bacterium was identified to species level using a combination of colony morphology, Gram staining and physicochemical tests. Isolates were stored in skimmed milk at −80 °C until use.

### Preparation of log phase bacteria

Glycerol stocks of log-phase bacteria were prepared as previously described [35]. Briefly, *S. oralis* strain ATCC 9811 was streaked onto blood agar plates and incubated aerobically (5% CO_2_, 18 h at 37 °C). Individual colonies were transferred into Todd-Hewitt Yeast medium (THY) and grown until stationary phase (5% CO_2_, 18 h at 37 °C). The cells were then inoculated into fresh THY and grown at 37 °C until an OD_600_ of ~0.6 was reached, corresponding to the log phase (approximately 5.0 × 10^8^ colony forming units (CFU)/mL) for ATCC 9811. Each 10 mL bacterial suspension was mixed with 2 mL of 80% glycerol, aliquoted into 1 mL microcentrifuge cryotubes, and stored at -80 °C. Samples for optical density, CFU, and mRNA measurements were withdrawn from the biofilm at 0, 3, 24, and 48 hours.

### Static biofilm model and growth-study using salivary MUC5B

MUC5B, purified from whole saliva using a CsCl gradient as previously described [12], was used to pre-coat a 24-well plate (NUNC, Denmark) with ~10 μg MUC5B and 3 mM Ca^2+^ (18 h, RT). Non-adhered MUC5B were removed before adding 1.25 × 10^8^ CFUs of thawed *S. oralis* ATCC 9811 glycerol stocks and an equal volume of Phosphate Buffered Saline (PBS) (pH 7). The bacteria were allowed to adhere to the MUC5B coating for 2 hours (5% CO_2_, 37 °C). Non-adhered cells were removed, while adhered cells were left to starve in PBS for 2 h (5% CO_2_, 37 °C). The metabolic phase was initiated by adding 10% THY ± 25% salivary MUC5B. Growth was monitored by measuring the OD_600_ and CFU at 0, 3, 24 and 48 hours. For CFU quantification, the supernatant in the well was used to gently disrupt and resuspend the biofilm, allowing collection of both the dispersed and surface-attached bacterial populations as a single, representative sample for plating. Samples for mRNA quantification were preserved using RNAprotect (QIAGEN, Germany). After centrifugation (5 min, 4000 × g), the cells were stored at −80 °C. Each well was only used once, and biofilm formation was conducted in biological triplicates. At time point 0, samples were collected immediately prior to the addition of MUC5B. As both control and test wells were identically coated and has not yet been exposed to MUC5B in the growth phase, these samples were biologically equivalent and served as a shared baseline for fold-change calculation, despite insufficient RNA yield for quantitative Reverse Transcription Polymerase Chain Reaction (RT-qPCR).

### MdpS and MdpS2 mRNA quantification using RT-PCR

RNA purification followed the ‘RNAprotect Bacteria Reagent Handbook - Protocol 5’ (QIAGEN). Briefly, cell pellets from biofilm growth were incubated in Tris-EDTA buffer (pH 8.0) with 15 mg/mL lysozyme (Sigma, United States) and 10% Proteinase K (QIAGEN) for 10 minutes at room temperature. Cells were disrupted in a TissueLyser II (QIAGEN) with glass beads at maximum speed (4 × 5 min). The supernatant was mixed with 80% ethanol and processed using the RNeasy Mini kit (QIAGEN) per ‘Protocol 7’. Samples were eluted in RNase-free water and RNA concentration was measured using the Qubit RNA BR Assay kit (Invitrogen, United States). cDNA was generated using the SuperScript IV VILO kit and protocol (Invitrogen) with 30 ng of template RNA. cDNA concentration was measured using the Qubit dsDNA HS Assay kit (Invitrogen) and stored at −20 °C until RT-PCR.

The RT-PCR was performed using the PowerTrack™ SYBR Green protocol (Thermo Fisher Scientific) with 5 ng cDNA and three primer pairs (Supplementary Table 1). Samples were analysed in biological and technical triplicates using QuantStudio 3 (v.2.7, Thermo Fisher Scientific). Relative mRNA levels of *mdpS* and *mdpS2* were calculated using the ΔΔCt method:

1. Subtract the Ct value of the housekeeping gene (*ef-tu*) from the target gene (*mdpS* or *mdpS2*): $∆\mathrm{Ct}={\mathrm{Ct}}_{\mathrm{target}}-{\mathrm{Ct}}_{h\mathrm{ousekeeping gene}}$
2. Subtract the Δ*Ct* of the control sample (THY) from the experimental sample (THY + MUC5B): $∆∆\mathrm{Ct}={∆\mathrm{Ct}}_{\mathrm{experimental}}-{∆\mathrm{Ct}}_{\mathrm{control}}$
3. Calculate the relative expression/fold change: $2^{-∆∆\mathrm{Ct}}$

### Cellular fractionation and localisation analysis

The subcellular localisation of MdpS2 was assessed by analysing three distinct fractions of planktonic *S. oralis* ATCC 9811: lysate (intracellular and cell-wall associated), surface-associated (papain-digested), and extracellular (culture supernatant). Sample preparation, proteolytic digestion, and Liquid Chromatography-Tandem Mass Spectrometry (LC-MS/MS) analysis were performed as previously described for MdpS [24]. Briefly, cells were grown in Brain Heart Infusion (BHI) broth, harvested, and fractionated. The lysate was obtained by sonication, the surface-associated fraction by papain digestion, and the extracellular fraction from the culture supernatant. Peptides were analysed using LC-MS/MS, and protein abundance was estimated based on total XIC intensities. Peptide identification was performed using MaxQuant against a custom *S. oralis* protein database including MdpS2: (GenBank: WP_000865736.1) tRNA threonylcarbamoyl adenosine modification protein YeaZ (Uniprot: J4UE30_STROR).

### Enzyme-induced biofilm dispersal

Biofilm pre-coating and adhesion were performed as described above for the static biofilm model. Non-adhered cells were removed before adding 50% THY to each well, allowing biofilm growth for 3 hours (5% CO_2_, 37 °C). Planktonic bacteria were removed, and wells were gently washed twice with PBS (pH 7.0) as previously described [35]. Enzyme pre-activation with MdpS, MdpS2 or TBS was conducted in 50 mM Tris (pH 7.0) with 2 mM Ca^2+^ using 25 μg enzyme per well. A 200 μL sample was added to cover the biofilm surface and incubated for 24 hours (5% CO_2_, 37 °C). Biofilm supernatants were transferred to microcentrifuge tubes for viable count determination (CFU/mL). The intact and attached biofilm were resuspended with 200 μL PBS (pH 7.0) for viable count determination. Biofilm dispersal was quantified by measuring viable bacterial counts from the supernatant (representing released cells) and from the remaining biofilm (after PBS resuspension). CFU was determined by serial dilution and plating on blood agar plates, followed by incubation at 37 °C for 24 hours. Dispersal was expressed as the ratio of supernatant CFUs to intact biofilm CFUs. This ratio was then normalised to the buffer-only control, which was set to 1. Each well was used once, and experiments were performed in technical triplicates.

### MUC5B sample preparation for nanoLC-MS analysis

Assessing potential hydrolytic preferences and differences between MdpS and MdpS2, 50 μg of pre-activated enzyme was employed in a reaction with ~50 μg MUC5B from whole saliva, prepared as previously described [12], in 2 mM Ca^2+^ in 50 mM Tris, pH 7.0 (24 h, 37 °C). A MUC5B control sample without enzyme was also included. All samples were reduced in 25 mM Dithiothreitol (DTT) (30 min, 60 °C), followed by alkylation in 62.5 mM iodoacetamide (20 min, 25 °C in the dark). The samples were acidified and desalted on Pierce Peptide Desalting C18 columns (Thermo Fisher Scientific) according to the manufacturer's instructions, followed by vacuum centrifugation to dryness.

As typical for CsCl gradient isolation of high-molecular-weight salivary mucins, secretory IgA may co-purify with MUC5B and was not intentionally added to the preparation. The identified hydrolytic sites on sIgA were not included in the manuscript. However, the mass spectrometry proteomics data can be found on the ProteomeXchange Consortium via the PRIDE [36] partner repository with the dataset identifier PXD062603.

### nanoLC-MS analysis of MUC5B hydrolysis

MUC5B (~25 μg) with or without enzyme digestion was reduced in 25 mM DTT (30 min, 60 °C), followed by alkylation in 50 mM iodoacetamide (20 min, RT, in the dark). The samples were cleaned from detergents with HiPPR Detergent Removal Resin (Thermo Fisher Scientific) and desalted on C18 columns (Pierce Peptide desalting spin columns) (Thermo Fisher Scientific) according to the manufacturer's instructions, followed by vacuum centrifugation to dryness.

NanoLC-MS/MS was performed on an Orbitrap Exploris 480 mass spectrometer (Thermo Fisher Scientific) interfaced with Easy-nLC1200 liquid chromatography system (Thermo Fisher Scientific). Peptides were trapped on an Acclaim Pepmap 100 C18 trap column (100 μm × 2 cm, particle size 5 μm) (Thermo Fisher Scientific) and separated on an in-house packed analytical column (75 μm × 35 cm, particle size 3 μm, Reprosil-Pur C18) (Dr. Maisch, Germany) using 90 minute runs with a gradient from 5 to 35% CAN in 0.2% formic acid at a flow of 300 nL/min. MS1 analysis range was *m/z* 350−2000 for peptides and *m/z* 600−2000 for glycopeptides, at a resolution of 120 K. MS2 analysis was performed in a data-dependent mode at a resolution of 30 K, using a cycle time of 2 seconds. The most abundant precursors with charges 2−7 were selected for fragmentation using Higher-energy Collisional Dissociation (HCD) at collision energy 30%. The isolation window was set to 2 *m/z* and the dynamic exclusion 30 s for peptides and 12 s for glycopeptides.

### Proteomic data analysis

The raw files were searched for identification using Proteome Discoverer version 2.4 (Thermo Fisher Scientific). The data were matched against human *Swissprot* database (May 2022) using Sequest as a search engine. The precursor mass tolerance was set to 5 ppm and fragment mass tolerance to 0.05 Da. Peptides consisting of at least 5 amino acid residues formed by unspecified hydrolysis were searched for. Variable modifications for methionine oxidation and fixed for carbamidomethylation were selected.

The raw files were searched for glycopeptides using rate (Protein Metrics, United States) within Proteome Discoverer version 2.4. The protein database was the human MUC5B sequence (Uniprot ID: Q9HC84) and the glycan databases were composed of 137 *N*-glycans and 48 *O*-glycans. The precursor mass tolerance was to 5 ppm and fragment mass tolerance to 30 ppm. Glycopeptide matches with Byonic scores above 300 in at least one of the datasets analysed in parallel were included. The MS data have been deposited to the ProteomeXchange Consortium via the PRIDE [52] partner repository with the dataset identifier PXD062603.

### MdpS and MdpS2-specific amino acid position weight matrices

Two sequence logos displaying the most probable amino acid ± 3 positions from the MdpS and MdpS2 hydrolytic sites were created using GraphPad 10 (v. 10.4.2, Graphpad Software Inc., United States). The hydrolytic data from 77 MdpS-specific and 95 MdpS2-specific high-score peptides generated from MUC5B hydrolysis were uploaded to Weblogo (v. 3.0, University of Berkeley). The data output specified the quantity of the amino acids at each specific position [1–6] and the numbers were entered into Excel and a probability percentage (%) was calculated.

### Rate-zonal centrifugation

Rate zonal centrifugation was conducted on 12 mL linear 10–35% sucrose gradients in PBS (pH 7.4), as previously described [37]. 500 μL samples were layered on top of the gradient and centrifuged in a Beckman L−90 ultracentrifuge (21,000 × g, 90 min, 15 °C) with a Beckman SW40 Ti swing-out rotor. Post centrifugation, the tubes were fractionated from the top, collecting 24 fractions. The pelleted material at the bottom was solubilized in 6 M urea. The sucrose concentration of each fraction was determined by measuring the refractive index. Fractions were then analysed for mucin distribution using immunodetection.

### Immunodetection

Following rate-zonal centrifugation and slot blot, immunodetection was performed as previously described [38]. Briefly, samples (50 μL) were slot blotted onto a nitrocellulose membrane using a Minifold II 72-well slot blot manifold (Cytiva). The membrane was rinsed in ddH_2_O, blocked (1 h, RT) with 5% (w/v) dried milk powder in PBS (pH 7.4), and washed in TBS with Tween (TBST). It was then incubated (12 h, 37 °C) with MAN-5BI rabbit polyclonal antiserum (1:2000 dilution) in TBST. After washing, the membrane was incubated in the dark with IRDye 800CW Donkey-Rabbit IgG (LI-COR Inc., United States) (1:10000 dilution) (1 h, RT). Following additional washes, secondary antibodies were visualised, and densitometry was performed using LI-COR Odyssey® CLx Infrared Imaging System (v. 2.2, LI-COR Inc.). The rolling average (three neighbouring fractions) of the MUC5B signal were calculated in relation to the total signal of each sample and were then plotted against the guanidine hydrochloride concentration calculated from the refractive index. The raw Relative Intensity Data (RID) were included to show the variations in the total signal between the samples.

### Particle tracking microrheology – BCi-NS1.1 culture and Viscoelastic effect of MdpS and MdpS2 in BCi-NS1.1 mucus

BCi-NS1.1 immortalised basal cell lines were provided by Ronald Crystal (Weill Cornell Medical College) and cultured based on previous methods [38,39]. BCi-NS1.1 cells with mucin 5B and mucin 5AC knockouts (MUC5B KO & MUC5AC KO) were generated as previously described[39]. Wild-type BCi-NS1.1, MUC5B KOs and MUC5AC KOs were cultured at air-liquid interface (ALI) at 37 °C, 5% CO_2_ on 12 mm transwell inserts (StemCell Technologies, Canada). The cells differentiated into pseudostratified mucociliary epithelium over the course of 4 weeks with media changed every other day. Mucus was collected from fully differentiated ALI cultures from the apical surface.

Particle tracking microrheology was performed as previously described [38]. Briefly, carboxylate-modified fluorescent polystyrene nanoparticles (100 nm) were coated with polyethylene glycol (PEG) via a carboxyl-amine linkage. Particle size and zeta potential were measured using Nanobrook Omni (Brookhaven Instruments, Sweden), resulting in diameters of 138.92 nm and near neutral surface charge. The diffusion of the PEG-coated nanoparticles (PEG-NP) in mucus gels was assessed using fluorescence video microscopy. Mucus (20 μL) were incubated (24 h, 37 °C) with: 100 mM Tris, 4 mM Ca^2+^ [pH 7.0]; Pre-activated MdpS in Tris-Ca^2+^ buffer; or Pre-activated MdpS2 in Tris-Ca^2+^ buffer. The mucus was then concentrated using a 10 kDa Amicon filter, and 20 μL of mucus was added to the microscopy chamber made from vacuum grease coated O-rings in which 1 μL of PEG-NPs were added and allowed to incubate for 30 min at RT. Videos were collected (33 Hz, 10 sec) using a Zeiss 800 LSM microscope at 63×-water immersion objective. Three independent samples were analysed per condition using ZEN Blue (v. 3.5, Zeiss), with at least three high-speed videos recorded per biological replicate. Particle tracking analysis was performed using a previously developed MATLAB (v. R2023b) algorithm [54]. Mean squared displacement (MSD) as a function of time lag (*τ*) was calculated for each particle as $\langle \mathrm{MSD}\left(\tau \right)\rangle \;=\;\langle \left(x^{2}+y^{2}\right)\rangle$. Based on the MSD, the generalised Stokes-Einstein relation was used to compute viscoelastic properties of the hydrogels. The Stokes-Einstein equation is, $G\left(s\right)=\frac{2k_{B}T}{\;(\pi \mathrm{as}\langle ∆r^{2}\left(s\right)\rangle )}$, where $k_{B}T$ is the thermal energy, *a* is radius of particle, and *s* is the complex Laplace frequency. The complex modulus (G*) was calculated as $G^{*}\left(\omega \right)=G^{’}\left(\omega \right)+G^{”}\;(i\omega )$, where *s* substitutes iω, with *i* being a complex number and ω being frequency. Finally, the pore size (*ξ*) of the mucus gel network was determined from Gʹ as: $\xi \;=\;{\left(\frac{k_{B}T}{G\prime }\right)}^{1/3}$.

## Results

### Conserved genomic structure of a potential *mdp* operon across *Streptococcus* species

A genomic study of *S. oralis* ATCC 9811 revealed that the genes encoding MdpS (Supplementary Figure 1) and MdpS2 (Supplementary Figure 2) are putatively located in the same operon, with a hypothetical protein annotated *in silico* located between them (Supplementary Figure 3). MdpS2 is positioned upstream of MdpS, as the nucleotides are transcribed from 3ʹ to 5ʹ. The operon's start and end positions were predicted using bioinformatics to identify sigma protein-binding sites, the terminator, the –35 and –10 promotor elements, and the transcription start site (TSS). Neighbouring genes are also annotated as hypothetical proteins, making it challenging to fully understand the genomic context of the ‘*mdp* operon’.

**Figure 3.**
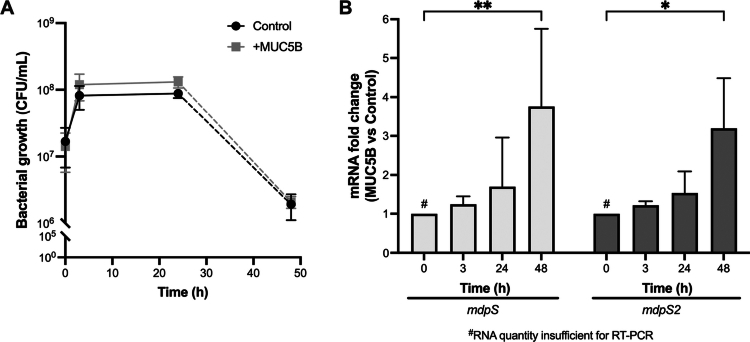
Temporal analysis of *S. oralis* biofilm growth and *mdpS/mdpS2* mRNA expression. (A) Bacterial growth (CFU/mL) of *S. oralis* ATCC 9811 grown on MUC5B-coated surfaces over 48 hours. Biofilm growth increased steadily over 3 hours and continued with a minor increase up to 24 hours, followed by both conditions experiencing a 98.5% reduction between 24 and 48 hours. A solid line is used between 3 and 24 hours due to consistent CFU/mL values across replicates, while a dashed line between 24 and 48 hours reflects the variability in the decline phase. (B) Relative mRNA expression of *mdpS* and *mdpS2* were quantified using RT-PCR using *ef-tu* as the reference gene, where expression was calculated using the ΔΔCt method. Data are shown as mean ± standard deviation from biological triplicates. Asterisks indicate significant differences (*p < 0.05, **p < 0.01) compared to the 0-hour time point. Statistical analysis was performed using two-way ANOVA with Dunnett's multiple comparison test. Time 0 samples were collected prior to MUC5B exposure and were biologically equivalent across conditions. Although the RNA yield was insufficient for RT-qPCR, this time point served as a shared baseline for fold-change analysis, with a set fold change of 1 reflecting equal mRNA levels in both conditions.

To further investigate the conservation of this operon across related *Streptococcus* species, the genomic context of the *mdp* operon was analysed using the BioCyc Genome Browser (Supplementary Figure 4). In all examined *S. oralis* strains, the operon structure and neighbouring genes were highly conserved, supporting the presence of a stable and potentially co-regulated gene cluster. In other *Streptococcus* species, including *S. sanguinis*, *S. gordonii,* and *S. constellatus,* the operon appears to be extended with additional downstream genes, suggesting species-specific adaptations or functional diversification. Despite these differences, the core positions of *mdpS* and *mdpS2,* annotated *in silico* as *tsaD* and *tsaB*, respectively, were preserved across all species analysed. Owing to the unavailability of the *S. oralis* ATCC 9811 genome in the BioCyc database, *S. oralis* ATCC 35037 was used as the reference for alignment.

### Conserved structural *N*-terminal domain despite low sequence identity between MdpS and MdpS2

To assess primary sequence conservation between MdpS and MdpS2, a multiple sequence alignment was performed using Clustal Omega and visualised in Jalview, in which the number of identical and high-scoring amino acids across defined regions were calculated. The highest similarity was observed in the *N*-terminal region (residues 1−100), where 25% of residues were identical to MdpS, and 47% showed high similarity, meaning residues with high to full conservation between included sequences (using Clustal Omega conservation symbols *, + , or 9) (Figure 1A, Supplementary Table 2). The C-terminal region (residues 101−227) showed lower conservation, with 17.3% identical residues and 28.3% high-scoring matches. Across the entire MdpS2 sequence, 20.7% of amino acids were identical to MdpS, and 36.6% were considered highly similar. These data suggest low primary sequence similarity in both the *N*- and C-terminal regions of the proteins.

The predicted tertiary structures of MdpS and MdpS2 generated via AlphaFold, were aligned in ChimeraX. Regions corresponding to amino acids 1−119 of MdpS, and 1−100 of MdpS2 were highlighted in light and dark green, respectively, to indicate similar domains (Figure 1B). A root-mean-square deviation (RMSD) value of 0 Å would reflect perfectly aligned folds. The RMSD for the full-length alignment (residues 1−227 in MdpS2) was 22.814 Å for all atom pairs and 0.924 Å for pruned pairs (excluding outliers). The green-coloured domain had an RMSD of 2.378 Å across all atom pairs and 0.940 Å for pruned pairs. The remaining region (residues 101−227), shown in black and white, had an RMSD of 20.376 Å across all atom pairs and 1.191 Å for pruned pairs. A summary of the sequence conservation and structural alignment data presented in Figure 1 is provided in Supplementary Table 2.

### MdpS2 exhibits enhanced NaCl and pH tolerance and higher substrate-specificity

The physicochemical properties of MdpS2 were assessed using etanercept under various conditions to determine optimal and tolerable parameters. Etanercept, an IgG1 fusion antibody, was selected as a substrate due to its glycan composition, which resembles that of MUC5B. Since enzyme kinetics are highly substrate-dependent, etanercept was considered a more biologically relevant alternative to conventional soluble substrates. MdpS2, which was pre-activated (24 h, 37 °C) in an activity buffer (2 mM CaCl_2_, 50 mM Tris (pH 7.0)) prior to substrate addition, exhibited optimal activity at pH 7.0, retaining high activity at both acidic and alkaline pH values of 6.0 to 9.0 (Figure 2A, Supplementary Figure 5). The effect of cations and EDTA on non-pre-activated enzyme revealed a clear dependency on Ca^2+^ and, to a certain extent, Mg^2+^, which facilitated autoproteolysis and subsequent self-activation (Figure 2B, Supplementary Figure 6). Further evaluation in the presence of increasing NaCl concentrations demonstrated that MdpS2 maintained a high hydrolytic activity across the tested range, indicating robust NaCl tolerance (Figure 2C, Supplementary Figure 7). MdpS2 activity was distinctly higher for mucin-like substrates, such as PSGL−1 and bovine fetuin, than for human immunoglobulins G1−4, E, and M (Figure 2D–E). However, a few faint bands, indicating partial hydrolysis of IgG3, IgG4, and IgE, were detected.

A comparative analysis of MdpS and MdpS2 was conducted to evaluate their rates of autoproteolysis/self-activation and enzymatic activity toward etanercept over time. MdpS2 appeared to self-activate more rapidly and demonstrated greater substrate hydrolysis at the 16- and 24-hour time points, as indicated by reduced intact substrate band intensity on SDS-PAGE (Supplementary Figure 8). However, it is important to note that the assay used an equal enzyme-to-substrate weight ratio, and since MdpS2 has a lower molecular weight than MdpS, this likely resulted in a higher molar concentration of MdpS2. This discrepancy could partially explain the apparent increase in activity, as more enzyme molecules were present per unit mass.

### *mdp* genes are expressed during *S. oralis* stationary phase biofilm growth

Bacterial growth (CFU/mL) was monitored for *S. oralis* ATCC 9811 growth on a MUC5B-coated plastic surface, where starved bacteria were grown in 10% Todd-Hewitt Yeast broth ± MUC5B during a 48-hour period (Figure 3A). Both the control and MUC5B samples exhibited a steady increase in biofilm growth after 3 hours. A further increase in the growth of both samples could be detected after 24 hours. Approximately 99% of the bacteria died between 24 and 48 hours in both samples. A continuous line is used in the graph between 3 and 24 hours because the CFU/mL values remained stable across this interval, indicating a plateau phase that was consistent across replicates. In contrast, the line between the 24- and 48-hour time points is dashed to indicate uncertainty in the shape of the decline, as the bacterial death phase was rapid and potentially variable despite multiple measurements being taken.

Combining the RT-PCR data, which measured the relative fold change of *mdpS* and *mdpS2* mRNA expression in the MUC5B sample compared to the control, revealed that neither of the genes was significantly upregulated during the log phase or in the early stationary phase at 3 to 24 hours (Figure 3B), although a gradual increase over time was noted for both genes. However, in a later stationary phase at 48 hours, both genes were significantly overexpressed in the presence of MUC5B. Although RNA from time 0 was insufficient for RT-qPCR, this did not affect the analysis, as both conditions were biologically identical at that point and served as a shared baseline for ΔΔCt calculations, with a set fold change of 1, indicating equal mRNA quantities in the control and MUC5B samples.

Breaking the average graphs into individual replicates revealed that minor changes in bacterial growth led to distinct differences in mRNA fold changes for both genes, especially in the 48 hour samples (Supplementary Figure 9). While all replicates displayed a similar pattern of expression over time, the highest and lowest values differed significantly, resulting in a high standard deviation (Figure 3B). As in Figure 3A, the lines between 24- and 48-hours in Supplementary Figure 9 are dashed to show possible shape variations. This variability may partly arise from differences in the MUC5B coating, despite standardised procedures and parallel processing of all samples. Minor inconsistencies, such as slight variations in the placement of the initial MUC5B droplet and rapid adhesion of MUC5B to the plastic surface, could introduce subtle differences in the initial coating, potentially influencing bacterial growth dynamics over time.

### MdpS2 is detected in the extracellular fraction of *S. oralis*

To assess the subcellular localisation of MdpS2, its distribution across lysate, surface-associated, and extracellular fractions was quantified. Analysis of the identified peptides and their corresponding total eXtracted ion current (XIC) intensities revealed that MdpS2 were present in all three fractions, with the highest signals observed in the surface-associated and extracellular samples (Supplementary Figure 10). The four included control proteins, ClpE, GroES, HtrA and DNA polymerase III alpha subunit, were detected in the subcellular fractions consistent with their expected localisation based on previous literature. The chaperones ClpE and GroES were found in both the lysate and surface-associated fractions. HtrA, a heat-shock-induced protease, was detected in the lysate and extracellular fraction, while DNA polymerase III alpha was identified exclusively in the lysate (Supplementary Figure 10).

### Bacterial release from *S. oralis* biofilms is promoted by MdpS and MdpS2

**Figure 4.**
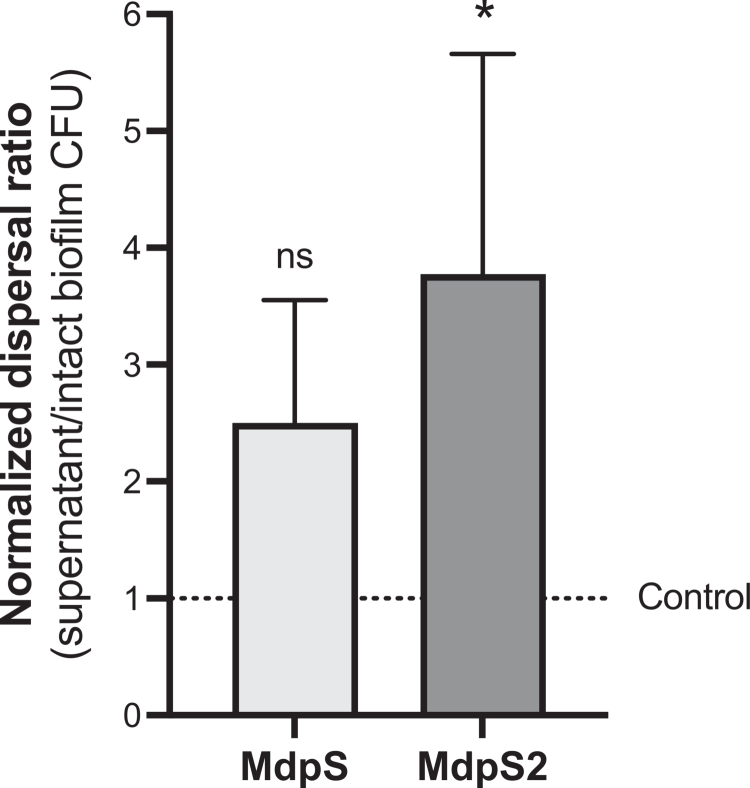
Enzyme-induced dispersal from *S. oralis* biofilms by MdpS and MdpS2. Biofilms of *S. oralis* ATCC 9811 were grown on MUC5B-coated surfaces and treated with MdpS, MdpS2, or buffer (control). After 24 hour treatment, viable counts (CFU/mL) were determined from both the supernatant (representing dispersed cells) and the intact (attached) biofilm, which was resuspended prior to plating. Dispersal was quantified as the ratio of supernatant CFUs to intact biofilm CFUs, normalised to the control (set to 1). Data are shown as mean ± standard deviation from technical replicates. Statistical analysis was performed using Kruskal Wallis ANOVA with Dunn's multiple comparison test (ns = *p *> 0.05, *p < 0.05).

To evaluate enzyme-induced biofilm dispersal, the ratio of viable bacteria in the supernatant (dispersed cells) versus the intact biofilm (attached cells) was measured following treatment with buffer (control), MdpS, or MdpS2 in a static biofilm model. The ratio was normalised to the control and set to 1, serving as a baseline for comparison. Both MdpS and MdpS2 increased the dispersal ratio, with mean values of 2.50 and 3.77, respectively (Figure 4). MdpS2 treatment resulted in a statistically significant increase (*p = 0.046) compared to the control, while MdpS did not (*p* = 0.138). High variability among technical replicates led to large standard deviations, which may be attributed to inconsistencies in creating a uniformly homogenous MUC5B coating prior to bacterial adhesion.

### Extensive and complementary hydrolysis of *N* – and C-terminal regions of salivary MUC5B by MdpS and MdpS2

**Figure 5.**
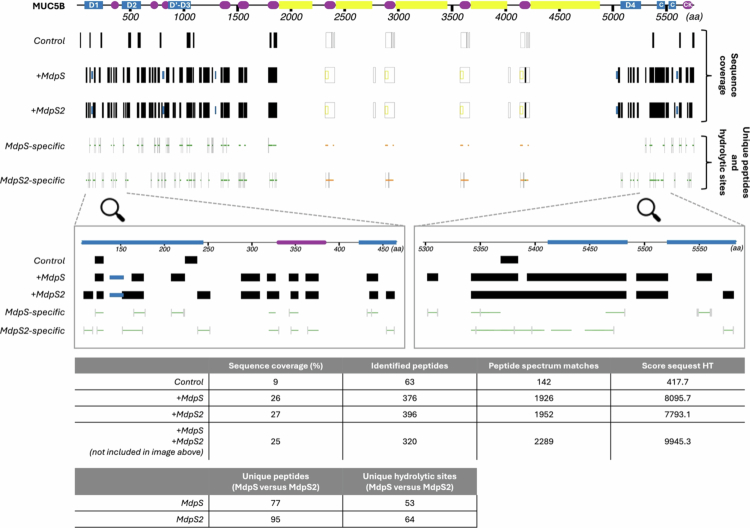
Degradation profile of salivary MUC5B by MdpS and MdpS2. Enzymatic degradation of MUC5B by MdpS and MdpS2 was analysed by nanoLC-MS/MS following a 24-hour incubation with pre-activated enzymes. The colours in the MUC5B illustration indicate key protein domains: blue – von Willebrand factor-like dimerisation domains with *N*-glycosylation sites (D1-D4, Dʹ, C domain), purple – cysteine-rich domains with abundant disulphide bridges (including CK domain), yellow - Pro/Ser/Thr-rich domains with numerous O-glycosylation sites. Filled black and grey boxes represent the sequence coverage and identified unique peptides, respectively. Hollow boxes with black (non-glycosylated peptides) or yellow (O-glycopeptides) borders indicate peptides with identical primary sequences at multiple sites, where precise localisation was not possible. Blue-filled boxes denote *N*-glycopeptides identified exclusively in the MdpS and MdpS2 samples. The number and width of the boxes in the sequence coverage map correspond to the extent and distribution of identified peptides across the MUC5B sequence. Increased box density and coverage in the *N*- and C-terminal regions of the MdpS and MdpS2 samples corresponded to higher peptide identification in these domains. This was reflected in the overall sequence coverage, which increased from 9% in the control to 26 and 27% for MdpS and MdpS2, respectively. Peptide mapping and hydrolytic site analysis, shown in the middle panel, revealed that MdpS2 generated 396 peptides, compared to 376 for MdpS. The enzymes exhibited overlapping hydrolysis patterns across MUC5B domains, as highlighted in the zoomed-in section. Samples treated with both proteases were excluded due to enzyme self-aggregation, which interfered with mass spectrometric analysis.

The proteolytic activity of MdpS and MdpS2 on salivary MUC5B was assessed by incubating untreated MUC5B from whole saliva with pre-activated MdpS and MdpS2 for 24 hours, followed by analysis using nanoLC-MS/MS (Figure 5). Both enzymes contributed to extensive degradation of both the *N*- and C-terminals, with sequence coverage of 26% for MdpS and 27% for MdpS2, compared to 9% for the control. However, MdpS2 led to the identification of 20 additional peptides (396) compared with MdpS (376), indicating a difference between them. Including both proteases in a reaction led to heavy enzyme self-aggregation and MS-data that were challenging to interpret; thus, it was excluded from the sequence coverage section of Figure 5.

By investigating their unique peptide matches and hydrolytic sites, potentially complementary functions between the enzymes were detected. Specific parts of both termini of MUC5B showed overlapping peptide coverage, highlighted by the zoomed-in sections (Figure 5). MdpS generated 77 unique peptides and 53 unique hydrolytic sites, showing minor preference for cysteine, aspartic acid, and glutamic acid around the hydrolytic site (Supplementary Figure 11). In contrast, MdpS2 generated 95 unique peptides and 64 unique hydrolytic sites with a slight preference for proline, glutamine, leucine, tyrosine, and aspartic acid.

### Structural integrity of MUC5B is differentially modulated by MdpS and MdpS2

**Figure 6.**
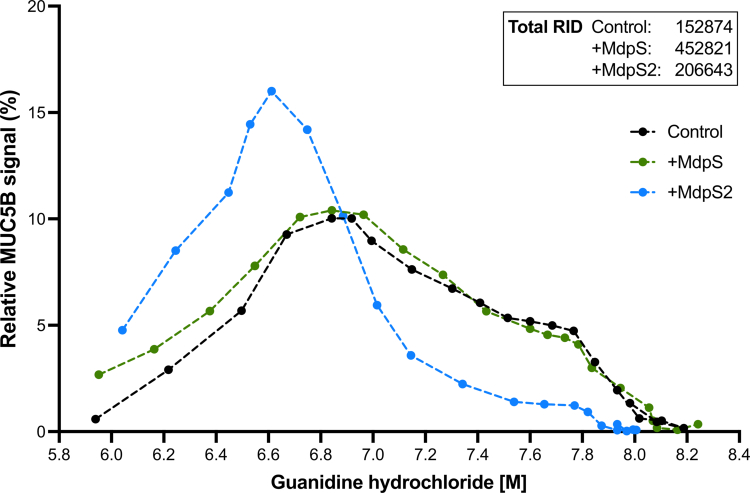
Sedimentation profile of MUC5B following enzymatic degradation. MUC5B samples treated with MdpS, MdpS2, or buffer (control) were separated using rate-zonal centrifugation and analysed using immunodetection. The relative signal intensity of each fraction was plotted against guanidine hydrochloride concentration, which reflects the sedimentation position. A shift in signal toward lower guanidine concentrations indicates a reduction in mucin size distribution and structural compactness. Total Relative Intensity Density (RID) values of each sample displayed in the upper right corner shows the large sample variations in the total MUC5B signal. The experiment was performed in a single replicate (*n* = 1).

The size distribution of MUC5B following incubation with MdpS and MdpS2 was assessed using rate-zonal centrifugation, followed by slot blotting and immunodetection. Identifying MUC5B with a signal at a lower guanidine hydrochloride concentration typically suggests a smaller size and less compact structure. MUC5B incubated with MdpS showed a higher percentage of the signal in the first four fractions than in the control, suggesting partial degradation of MUC5B (Figure 6). The MdpS2 sample demonstrated a change in the sedimentation profile of MUC5B, with a marked increase in MUC5B fractions towards the top of the gradient (lower guanidine hydrochloride concentrations), indicating a substantial reduction in the size of MUC5B. The analysis was performed with a single replicate. The total Relative Intensity Density (RID) signal of each sample revealed an almost three-fold increase in the MdpS sample, whereas the MdpS2 incubation had a 35% higher RID compared to the control.

### MdpS and MdpS2 modify the properties of regular - and MUC5B or MUC5AC knockout BCi-NS1.1 mucus gels

**Figure 7.**
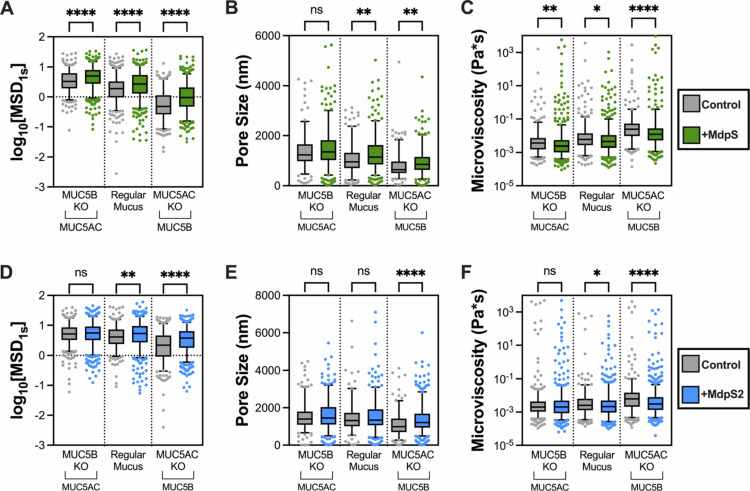
Alteration of mucus gel properties by MdpS and MdpS2. Particle-tracking microrheology was used to assess the impact of MdpS and MdpS2 on the rheological properties on mucus extracted from BCi-NS1.1 cultures, including wild-type (regular), MUC5AC knockout (KO), and MUC5B KO mucus. Three parameters were measured to evaluate mucus structure and flow properties: (A, D) Mean squared displacement (MSD) reflects nanoparticle mobility and is inversely related to mucus viscosity; (B, E) Pore size estimates the average spacing with the mucus mesh, derived from the elastic modulus; (C, F) Microviscosity quantifies the resistance to flow within the mucus gel. Each panel represent biological replicates shown as box-scatter plots. Boxes indicate the interquartile range (25−75%), with medians marked as a horizonal lines. Whiskers extend to the 5th and 95th percentiles, where individual points are staggered within each group to improve visibility. Statistical analysis was assessed using Kruskal–Wallis ANOVA with Dunn's multiple comparisons test (ns = *p *> 0.05, *p < 0.05, **p < 0.01, ****p < 0.0001).

Microrheology measurements demonstrate the effects of MdpS and MdpS2 on the biophysical properties of mucus extracted from BCi-NS1.1 air-liquid interface cultures, including regular mucus, MUC5AC knock-out (KO) mucus or MUC5B KO mucus. The mean squared displacement (MSD) data, plotted on a logarithmic scale, revealed a significant increase in nanoparticle diffusion through all mucus types for MdpS treated groups (****) (Figure 7A). Since MSD reflects the average distance particles travel over time, this increase indicates a reduction in mucus viscoelasticity, allowing particles to move more freely. However, the effective increase in pore size, which estimates the average spacing within the mucus mesh, was only significant in regular (**) and MUC5AC KO (MUC5B) (**) mucus (Figure 7B). This suggests that MdpS treatment loosens the mucus network in these conditions, potentially enhancing permeability. Microviscocity, derived from the viscoelastic modulus, was significantly decreased, indicating a reduction in the mucus' resistance to flow. Variations were observed among different mucus types: MUC5B KO (MUC5AC) (**); regular (*); MUC5AC KO (MUC5B) (****) compared to controls (Figure 7C). These changes reflect a shift towards a more fluid-like behaviour in the mucus matrix. In contrast, MdpS2 had no significant effect on MUC5B KO (MUC5AC) mucus in any experiment but significantly impacted the properties of MUC5AC KO (MUC5B) mucus in all tests (****) (Figure 7D–F). The effect of MdpS2 on regular mucus was only observed in the MSD (**) and microviscocity (*) experiments. The number of data points varied between experiments, resulting in fluctuations in the number of individual points in the graph indicating the 0−5% and 95−100% outliers (Supplementary Table 3).

## Discussion

In this study, we characterised two homologous proteases, MdpS and MdpS2, that are putatively encoded within the same operon in *S. oralis* ATCC 9811. Although these enzymes share limited sequence identity, structural modelling revealed conservation of tertiary structure in a domain that may be functionally relevant. Comparative biochemical and biofilm assays demonstrated that MdpS2 exhibited greater tolerance to environmental stressors, faster autoproteolytic activation, and stronger biofilm dispersal capabilities than MdpS. Both enzymes contributed to the complementary and extensive hydrolysis of salivary MUC5B, thereby modulating its structural integrity and rheological properties. These findings suggest that MdpS and MdpS2 may act in concert to remodel mucosal environments, supporting bacterial adaptation under nutrient-limited conditions and potentially influencing *S. oralis* colonisation dynamics and host interactions.

To begin exploring the biological relevance of MdpS and MdpS2, we first examined their expression dynamics to understand how *S. oralis* regulates these enzymes during biofilm growth and nutrient limitation. Both genes were significantly upregulated during late stationary-phase biofilm growth on MUC5B (Figure 3B), suggesting a starvation-induced expression pattern. This aligns with bacterial strategies where stress-responsive enzymes, including proteases, glycosidases, and transporters, are activated to promote dispersal and nutrient acquisition. For instance, in *Vibrio cholerae*, the haemagglutinin protease (HAP) is induced under HapR and RpoS control during the stationary phase, facilitating detachment and environmental adaptation [40]. In our static biofilm model, *S. oralis* did not exhibit a significant increase in bacterial load, typically observed as a >10-fold increase, when grown with MUC5B (Figure 3A). This outcome is consistent with previous findings that complete mucin degradation requires enzymatic cooperation among multiple bacterial species [22]. Although no single species can fully hydrolyse MUC5B, consortia of supragingival plaque bacteria have demonstrated this capability, emphasising the importance of bacterial cooperation in mucin degradation. Nevertheless, both MdpS and MdpS2 were able to hydrolyse MUC5B extensively without enzymatic pre-treatment (Figure 5), suggesting that their expression may serve roles beyond nutrient acquisition through carbon and nitrogen source sequestration – such as biofilm remodelling and dispersal.

Given their starvation-induced expression and regulated activation, Mdp proteases appear to play a strategic role in mucosal adaptation, supporting bacterial survival under nutrient-limited conditions. The expression pattern, combined with the enzymes' ability to modulate mucus structures and properties (Figures 6 and 7), points to a broader ecological function for Mdp proteases in facilitating bacterial migration and re-colonisation of oral niches more conducive to survival. Both enzymes are synthesised as zymogens requiring autoproteolytic activation (Supplementary Figure 8), a process that may be regulated by host- or microbe-derived factors [41]. Such multi-level regulation likely ensures that mucin degradation occurs only under appropriate conditions, preserving the protective integrity of the mucus barrier while enabling strategic bacterial release. These features align with starvation-responsive proteolytic systems and reinforce the potential role of MdpS and MdpS2 in environmental adaptation. Furthermore, the conserved genomic positioning of *mdpS* and *mdpS2* across *Streptococcus* species (Supplementary Figure 4) underscores the evolutionary importance of tightly regulating their expression, suggesting that these enzymes play a stable and functionally relevant role with the oral streptococcal ecosystem.

Building on the observed expression and regulatory features of MdpS and MdpS2, it was examined whether they could induce biofilm dispersal – a key process in oral microbial ecology. A baseline value of 1 in the dispersal assay reflected the passive release of cells under buffer-only conditions, whereas values above 1 indicated enzyme-induced dispersal (Figure 4). Such an increase would likely result from a Mdp-mediated disruption of the MUC5B coating or biofilm matrix, leading to cell release into the supernatant. As dispersal is critical in oral biofilm ecology – allowing bacteria to adapt to dynamic salivary flow, colonise fresh niches, and contribute to microbial succession during biofilm maturation [42] – such enzymatic activity could enable recolonisation of new surfaces under nutrient-limited conditions. Notably, MdpS2 exhibited a higher dispersal ratio compared to MdpS (Figure 4). Although this difference was not statistically significant, it suggests that MdpS2 may play a more prominent role in biofilm disruption. Other enzyme-induced dispersal mechanisms exists in *Pseudomonas aeruginosa*, where enzymes such as cellulase, xylanase, and *α*-amylase degrade biofilm matrices in response to environmental signals, including nutrient availability [42,43]. Using a similar enzyme-induced biofilm model, proteases such as HtrA, trypsin, proteinase K, and papain were shown to induce bacterial release from *Streptococcus pneumoniae* biofilms [44]. Supporting their proposed role in biofilm dispersal, both MdpS and MdpS2 were analysed and detected as secreted enzymes (Supplementary Figure 10) [24] indicating their potential to mediate mucin modification and promote bacterial release. These parallels suggest Mdp enzymes contribute to a broader bacterial strategy for biofilm remodelling and dispersion. These findings have significant implications for oral health, as biofilm dispersal promotes bacterial colonisation of new surfaces and may serve as a key mechanism underlying *Streptococcus* species predominance in the oral microbiome and their influence on bacterial succession. Moreover, understanding biofilm dispersal mechanisms could provide insight into *Streptococcus* species' ecological success and represent a novel therapeutic strategy for managing biofilm-associated infections, an area with considerable clinical need [45].

To better understand how Mdp enzyme activity contributes to biofilm dispersal, we examined their effects on the structural integrity of the MUC5B network. Understanding how this enzymatic activity disrupts biofilms requires insight into the mechanisms by which MUC5B maintains its cohesive and viscoelastic properties under physiological conditions. Upon granular release, MUC5B rapidly expands into a net-like structure of densely packed mucin fibres connected by protein-rich nodes, forming a viscoelastic gel that regulates microbial communities and preserves mucosal barrier function [46]. The stability of this network is governed by ionic interactions, with sodium and calcium playing key regulatory roles [47,48]. MUC5B polymers are initially formed through disulphide-linked C-terminal dimerisation, followed by further polymerisation via *N*-terminal multimerization (D1-3 domains) [49], which finalises the network assembly. Interestingly, the cysteine residues within the D3 domain, which appear critical for these polymeric interactions [50], are hydrolysed at four sites by both Mdp enzymes out of a total of 17 hydrolytic sites in the D3 domain (Figure 5). This extensive hydrolysis may explain the substantial structural disruption detected in the immunodetection assay following MUC5B sedimentation (Figure 6). While MdpS hydrolysis did not result in a distinct distribution shift of MUC5B, the pronounced shift observed with MdpS2 may be attributed to the 64 unique hydrolytic sites, which have a larger structural impact than those of MdpS. Although MdpS did not cause a prominent shift in MUC5B distribution, the marked increase in the total RID signal suggests that MdpS sufficiently modifies the structure to alter the binding of the primary antibody to its epitope. The lower total signal of MdpS2 compared to MdpS could be due to the four additional hydrolytic sites (Figure 6) just *N*-terminal to the binding region of the primary antibody [51], possibly affecting accurate epitope recognition. These additional sites could further explain the greater structural impact of MdpS2 hydrolysis on MUC5B, as they are located within the cysteine-rich domains interspersed throughout the central O-glycan-rich mucin region (Figure 5). These highly conserved domains may play a critical role in regulating mucus network properties through non-covalent cross-links between mucin polymers [52,53].

Beyond structural modulation, the specialised substrate specificities and environmental sensitivity further define their functional divergence and therapeutic relevance. Microrheological analyses further demonstrated that MdpS2 exhibited specificity for MUC5B, whereas MdpS hydrolysed both major airway mucins, MUC5B and MUC5AC (Figure 7). Despite their similar amino acid sequences, the structural differences between MUC5B and MUC5AC likely account for this substrate selectivity [54,55]. Given that *S. oralis* primarily colonises the oral cavity, it has likely evolved Mdp enzymes optimised for MUC5B degradation, as supported by physicochemical analyses and substrate specificity assays (Figure 2) [24]. MdpS, which is more sensitive to changes in pH and sodium concentration, exhibits a broader substrate range beyond mucins, which may explain its ability to hydrolyse MUC5AC in the present study. In contrast, MdpS2 was highly specific to MUC5B. The impact of mucus rheology is particularly significant in conditions such as cystic fibrosis, where the accumulation of thick, cross-linked MUC5B and MUC5AC impairs mucociliary clearance and exacerbates infection and inflammation [14]. In this context, targeted MUC5B degradation by MdpS2 could alleviate mucus plugging, potentially without compromising lung defence, offering a novel therapeutic avenue alongside traditional mucolytic agents, such as dornase alfa (Pulmozyme®). In contrast, asthma and chronic obstructive pulmonary disease (COPD) primarily involve airway inflammation rather than excessive mucus cross-linking [56]. In the oral cavity, altered mucus rheology influences bacterial aggregation and dispersal in oral biofilms. Increased rigidity promotes biofilm maturation, whereas reduced viscosity facilitates microbial dispersal and recolonisation, potentially shifting the microbial composition and the balance between commensal and pathogenic species. Thus, Mdp enzymes not only offer therapeutic potential for chronic respiratory conditions but may also play a key role in modulating the balance of microbial communities in the oral cavity. MdpS, with its broader substrate specificity, may help reduce mucus accumulation and restore the balance of MUC5B and MUC5AC, which is essential for effective mucociliary clearance. The MUC5B-specific activity of MdpS2 suggests that it could be particularly useful for targeted interventions in cystic fibrosis.

Finally, structural analysis revealed that MdpS and MdpS2 diverge from canonical protease families, suggesting evolutionary specialisation for mucosal environments. Interestingly, neither MdpS nor MdpS2 includes a predicted secretion signal or known cell wall anchor motif, and no conserved catalytic residues could be identified based on sequence similarity to known proteases. Furthermore, neither enzyme shares domain homology with well-characterised protease classes. MdpS2 shows moderate sequence conservation with two actin-like ATPase domains and exhibits some overall homology (28%) to the characterised *E. coli* tRNA threonylcarbamoyladenosine biosynthesis protein TsaB, whereas MdpS aligns partially (44%) with a characterised *E. coli* TsaD [24]. These associations, though limited, suggests a possible evolutionary relationship to atypical proteases and point to a divergence from classical protease families. The low RMSD observed between the *N*-terminal domains (Figure 1), supports that both enzymes belong to the same atypical and novel protease family. However, whether the active site or substrate-binding region resides within this shared domain is currently unknown. The atypical domain composition may reflect specialised enzymatic functions adapted to mucosal environments and underscores the importance of experimental characterisation, as reliance on generic *in silico* annotations may overlook novel proteolytic functions.

In summary, our findings provide new insights into the functional roles of MdpS and MdpS2 in *S. oralis* adaptation to mucosal surfaces, which is supported by their expression as part of a starvation response. The distinct substrate specificities and functional capabilities of these proteases highlight their complementary roles in biofilm formation and dispersal, as well as their potential to modulate host mucus structures. Their differing pH optimum ranges and NaCl sensitivity further reinforce this complementarity, enabling enzymatic activity across a broader range of environmental conditions. Similar patterns of overlapping but distinct pH profiles have been observed in other enzyme groups [57] and are thought to enhance bacterial adaptability and resilience. MdpS, with its broader substrate range and sensitivity to environmental factors, may contribute to general mucus remodelling, whereas MdpS2, with its specificity for MUC5B and higher activity in mucus modulation, appears particularly suited for targeted biofilm disruption. These properties open up potential therapeutic applications, especially in diseases such as cystic fibrosis, where both proteases could offer a novel strategy for alleviating mucus plugging without compromising mucosal defence. Moreover, the differential expression of these proteases suggests a finely tuned response to environmental cues, such as pH and cell density, which could be key to the success of *S. oralis* in colonising and interacting with mucosal surfaces. Together, these results underscore the complex interplay between bacterial adaptation and host interactions, highlighting the potential for therapeutic interventions targeting biofilm dynamics. Additionally, they emphasised the possible role of Mdp proteases as mucolytic agents in modifying mucus rheology in chronic respiratory conditions.

Despite the strength of our findings, limitations should be noted. This study primarily investigates MdpS and MdpS2 using recombinant proteins *in vitro* and thus cannot directly confirm their functional activity within the native *S. oralis* cellular context. While we observed clear upregulation of both genes during stationary-phase biofilm growth – a biologically relevant condition that mimics nutrient limitation – we did not assess enzyme function through bacterial mutants or *in vivo* models. Therefore, although our biochemical and expression data suggest these enzymes play a role in mucin degradation and biofilm remodelling, definitive evidence for their function in the oral environment requires future studies using gene knockouts, complemented strains, or proteomic analyses of native biofilms.

## Conclusions

Understanding how oral bacteria interact with host mucins is essential to unravel the mechanisms that govern microbial colonisation, persistence, and dispersal at mucosal surfaces. This study identified and characterised MdpS2 and, together with the previously described MdpS, demonstrates the roles of two distinct proteases in *Streptococcus oralis* that degrade the salivary mucin MUC5B and modulate biofilm behaviour. By characterising their structural and functional differences, we revealed a complementary enzymatic system that supports bacterial adaptation under nutrient-limited conditions and promotes biofilm dispersal. These findings shed light on how early colonising streptococci could influence the structure and dynamics of oral biofilms, contributing to microbial succession and ecological balance. Moreover, the ability of these enzymes to alter mucus rheology suggests their potential applications in developing targeted mucolytic strategies for managing biofilm-associated infections and mucus-related disorders. This work advances our understanding of streptococcal behaviour in the oral cavity and highlights the importance of mucin-degrading enzymes in host-microbe interactions.

## Ethical approval

This study did not involve human participants, animals, or sensitive data, and therefore did not require ethical approval.

## Supplementary Material

Supplementary material Supplementary Information

## Data Availability

The datasets presented in this study can be found in online repositories. The names of the repository/repositories and accession number (s) can be found at: https://www.ebi.ac.uk/pride/archive/, PXD062603.
