# Supplementary material for: Functional divergence of MdpS and MdpS2 reveals mucin-targeting strategies in Streptococcus oralis
Source: J Oral Microbiol. 2025 Oct 26;17(1):2571186. doi: 10.1080/20002297.2025.2571186 (PMC12557822; doi:10.1080/20002297.2025.2571186)
Supplement: Supplementary material — Supplementary Information [file ZJOM_A_2571186_SM9033.docx]

***Supplementary Information***

**Functional divergence of MdpS and MdpS2 reveals mucin-targeting strategies in *Streptococcus oralis***

**Fredrik Leo^1,2*^, Jonas Nilsson^3^, Liisa Arike^3^, Sahana Kumar^4^, Emma Hilton^5^, Rolf Lood^2,6^, David J Thornton^5^, Gregg A Duncan^4^, Gunnel Svensäter^1^, Claes Wickström^1^**

*****Address correspondence to Fredrik Leo, [fredrik.leo@mau.se](mailto:fredrik.leo@mau.se)

This supplementary file provides additional data supporting the characterization of the mucin-degrading proteases MdpS and MdpS2 from *Streptococcus oralis*. Included are FASTA sequences of the recombinant enzymes, primer details for RT-PCR, genomic context analyses, and comparative structural data. Representative gels illustrate the physicochemical properties and substrate specificity of MdpS2, while time-course assays compare enzymatic activity between MdpS and MdpS2. Additional figures present individual biofilm replicates, subcellular localization data, and enzyme-specific hydrolytic site preferences.

>MdpS

MKDRYILAFETSCDETSVAVLKNDDELLSNVIASQIESHKRFGGVVPEVASRHHVEVITACIEEALAEAGITEEDVTAVAVTYGPGLVGALLVGLSAAKAFAWAHGLPLIPVNHMAGHLMAAQSVEPLEFPLLALLVSGGHTELVYVSEAGDYKIVGETRDDAVGEAYDKVGRVMGLTYPAGREIDELAHKGQDVYDFPRAMIKEDNLEFSFSGLKSAFINLHHNAEQKGESLSKEDLSASFQAAVMDILMAKTKKALGEYPVKTLVVAGGVAANKGLRERLAAEITDVKVIIPPLRLCGDNAGMIAYASVSEWNKGNFAGLDLNAKPSLAFDTMEGSGHHHHHH

**Supplementary figure 1**: FASTA sequence of recombinant MdpS (GenBank accession number WP_084852800.1) including a C-terminal GSG linker and 6xHis-tag.

**Supplementary figure 2**: FASTA sequence of recombinant MdpS2 (GenBank accession number WP_139689688.1) including a C-terminal GSG linker and 6xHis-tag.

>MdpS2
MKVLAFDTSSKALSLAILEDKQVLAETMINIKKNHSITLMPAIDFLMASLDLTPKDLDNGPGSYTGLRIAVATAKTLAHTLKIELVGVSSLLALIPKQVEGLVIPVMDARRNNVYAGFYENAQSVFPEAHLSFAEVLEQVKNAEQVTFVGEVGAFVEQIQEHLPQASYQETLPNAANLALWAWDKKAASLHDFVPNYLKRVEAEENWLKNHTESGESYIKRLGSGHHHHHH

**Supplementary Table 1:** Detailed Primer Information for RT-PCR Measurements.

| **Primer name** | **Sequence  (5’ to 3’)** | **Target gene** | **Product  Size (bp)** | **Annealing  Temperature (°C)** | **GC  Content (%)** |
| --- | --- | --- | --- | --- | --- |
| Elongation factor Tu – F | GGACGTATCGACCGTGGTAC | *ef-tu* | 185 | 65.3 | 60.0 |
| Elongation factor Tu – R | CCACGTTCGATTTCGTCACG |  |  |  | 55.0 |
| MdpS – F | GACGACCAGCTCTTGTCCAA | *mdpS* | 102 | 66.0 | 55.0 |
| MdpS – R | AACCTCGACATGGTGACGAC |  |  |  | 55.0 |
| MdpS2 – F | TTGTCGGAGAAGTTGGAGCC | *mdpS2* | 126 | 66.1 | 55.0 |
| MdpS2 – R | AGGAGGCTGCTTTCTTGTCC |  |  |  | 55.0 |

***

***

***Supplementary figure 3:*** **Genomic organization of *mdpS* and *mdpS2* in *S. oralis* ATCC 9811.** The genomic overview highlights the positions of the *mdpS* (green) and *mdpS2* (blue) in relation to neighboring genes, all of which are annotated *in silico* as hypothetical proteins. The predicted binding site for the sigma (σ) factor and the terminator are indicated with grey and red circles, respectively. The positions of the -35 and -10 promotor elements are marked with grey circles, while the predicted transcription start site (TSS) is depicted with a green circle.

**”*mdp* operon” in *S. oralis* ATCC 9811**


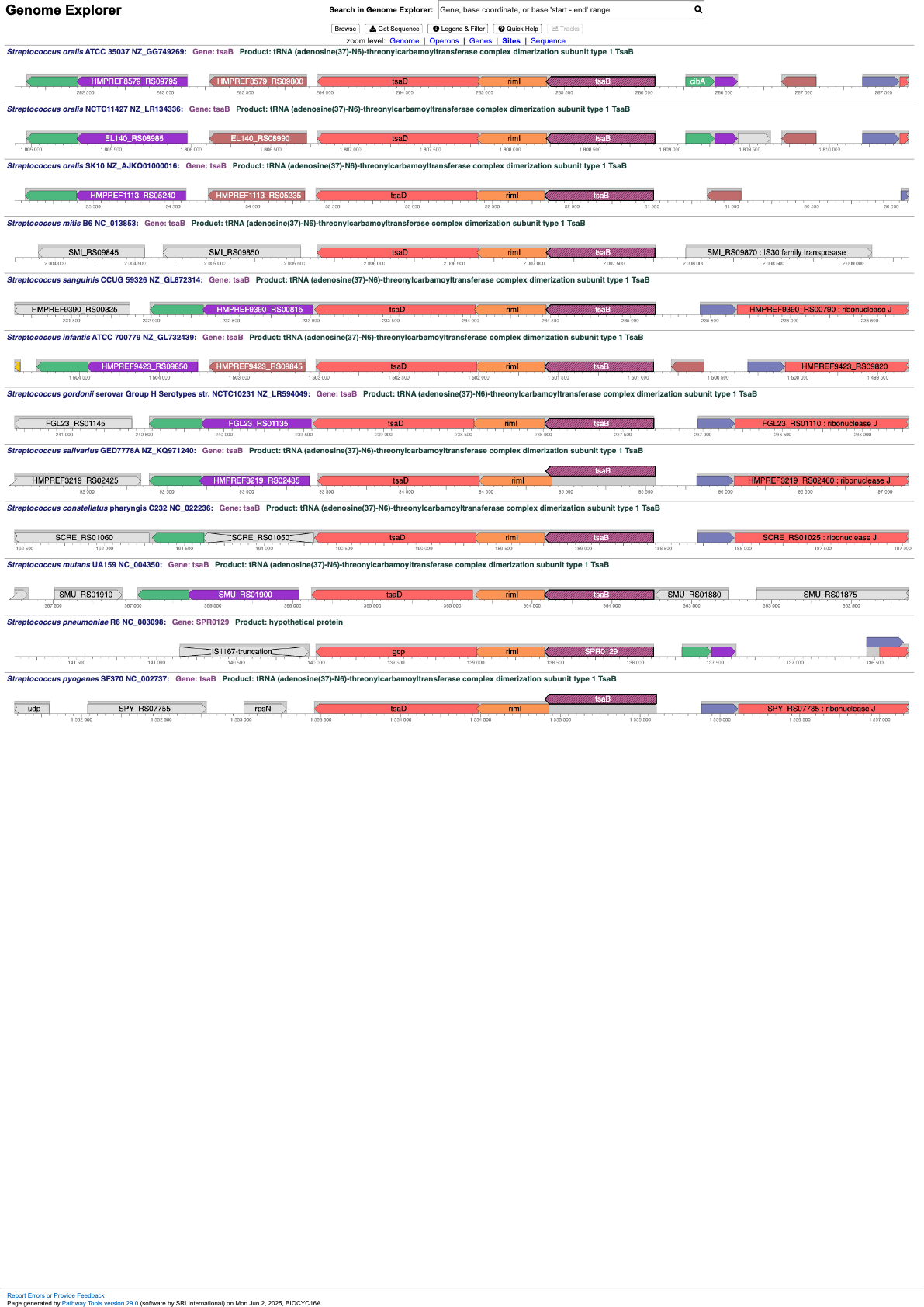


**Supplementary figure 4: Comparative genomic alignment of the mdp operon region across selected Streptococcus species.** The genomic context of the mdp operon was analyzed using the BioCyc Genome Browser, comparing orthologous regions across selected Streptococcus species. In the alignment, mdpS and mdpS2 genes are annotated in silico as tsaD and tsaB, respectively, due to partial similarity with characterized E. coli homologues. In S. oralis strains, the operon structure and neighboring gene context appears highly conserved. In other Streptococcus strains, the alignment reveals conserved gene position with the putative mdp operon, although the operon appears extended in S. sanguinis, S. gordonii, and S. constellatus. S. oralis ATCC 35037 was used as the reference strain due to unavailability of the ATCC 9811 genome in BioCyc. Genes with identical colors across strains indicate conserved function. Genes highlighted with a grey background are predicted to belong to the same operon, suggesting potential co-transcription and functional relatedness.

**Supplementary Table 2: Summary of the sequence and structural similarity between MdpS2 and MdpS across defined regions.** The number and percentage of amino acids in MdpS2 that are either identical (*) or show high similarity (*, +, 9) to those in MdpS are based on the multiple sequence alignment shown in Figure 1A. Structural similarity was assessed using ChimeraX. Root-mean-square deviation (RMSD) values (in Å) were calculated for all atom pairs as well as for pruned pairs (excluding outliers), with lower values indicating higher structural similarity.

| **MdpS2 region** | **# Identical (*) amino acids (%)** | **# High score (*, +, 9) amino acids (%)** | **RMSD for all atom pairs (Å)** | **RMSD for pruned atom pairs (Å)** |
| --- | --- | --- | --- | --- |
| 1-100 | 25 (25.0%) | 47 (47.0%) | 2.378 | 0.940 |
| 101-227 | 22 (17.3%) | 36 (28.3%) | 20.376 | 1.191 |
| Complete sequence (1-227) | 47 (20.7%) | 83 (36.6%) | 22.814 | 0.924 |

**Supplementary figure 5:** A representative SDS-PAGE analysis of MdpS2 activity across a pH range. Etanercept was used as the substrate.

**Supplementary figure 6:** A representative SDS-PAGE analysis of MdpS2 activity in the presence of divalent cations (Ca^2+^, Mg^2+^, Zn^2+^) and EDTA. Etanercept was used as the substrate.

**Supplementary figure 7:** A representative SDS-PAGE analysis of MdpS2 activity under increasing NaCl concentrations. Etanercept was used as the substrate.

**Supplementary figure 8: Comparison of MdpS and MdpS2 activity over time.** SDS-PAGE analysis shows the hydrolysis of etanercept by MdpS and MdpS2 at different time points (2, 6, 16, 24 h). The molecular weight markers (kDa) are indicated on the left. The etanercept control is located in the first lane, while non-incubated enzyme controls are in the right-most lanes. The bands corresponding to the intact enzyme are clearly visible, indicating that autoproteolysis over time can also be detected.


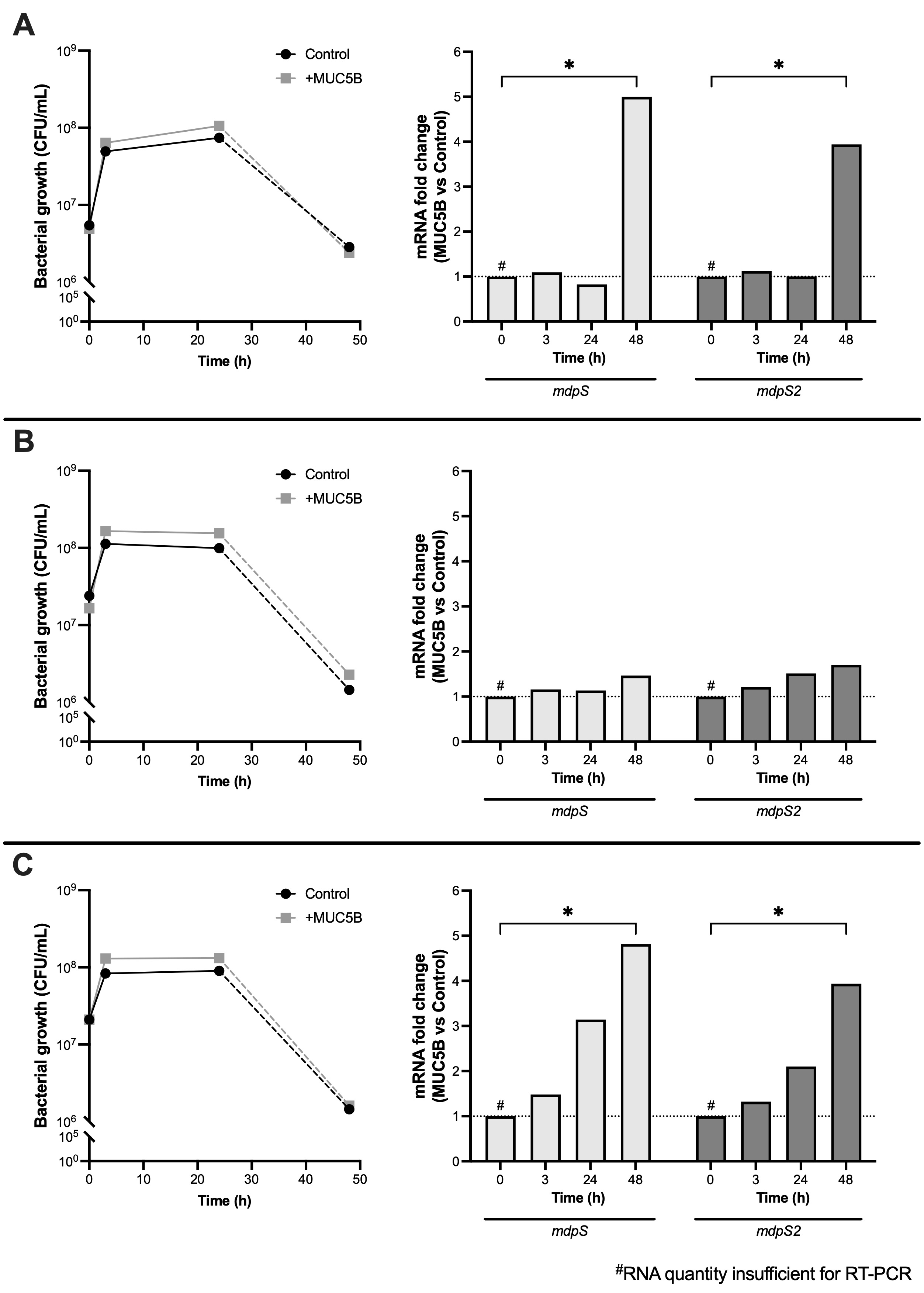


**Supplementary figure 9: Individual replicates showing S. oralis biofilm growth and relative mdpS and mdpS2 mRNA expression over time.** The left panels show the bacterial load (colony-forming units (CFU)/mL) in S. oralis biofilms over time for control and MUC5B samples. The exact CFU/mL during the stationary phase between 24 and 48 hours was not determined, and it is thus marked with a dashed line. The right panels display the relative mRNA fold change for mdpS and mdpS2 in the presence MUC5B compared to control at different time points (0, 3, 24, 48 h), using the housekeeping gene ef-tu as the reference. Asterisks indicate significant differences (*p < 0.05) compared to the 0-hour time point. The 0-hour time point was set to a fold change of 1 due to insufficient RNA amounts for both control and MUC5B samples. Panel A, B, and C corresponds to biological replicate 1, 2, and 3, respectively. Statistical analysis was performed using two-way ANOVA with Šídák’s multiple comparisons test. The primers used for the RT-PCR experiments can be found in Supplementary Table 1.

**Supplementary figure 10: Subcellular localization of MdpS2 in S. oralis ATCC 9811. (A)** Quantification of MdpS2 and control proteins in lysate, surface-associated, and extracellular fractions based on the eXtracted ion current (XIC) intensities of all isotopic clusters corresponding to identified peptides. **(B)** Number of unique peptides identified for each protein. Control proteins with known localization were included for validation.


***Supplementary figure 11:*** **Enzyme-specific amino acid specificity analyzed by nanoLC-MS.** Position Weight Matrices were constructed to illustrate the unique hydrolytic sites of MUC5B targeted by **(A)** MdpS and **(B)** MdpS2. The y-axis position of each letter represents the probability of a particular amino acid occurring at a specific position, three residues away from the hydrolytic site (indicated by the dashed line). The matrices are based on the 77 unique peptides for MdpS and 95 unique peptides for MdpS2. Amino acids are color-coded according to their side chain properties: green for positively charged, red for negatively charged, yellow for uncharged, purple for special cases, and blue for hydrophobic.

**Supplementary Table 3:** Overview of data points collected in individual microrheology experiments.

| **Experiment** | **Control: #data points MdpS experiments** | **+MdpS:  #data points** | **Control: #data points MdpS2 experiments** | **+MdpS2:  #data points** |
| --- | --- | --- | --- | --- |
| log10 MSD:  MUC5B KO | 353 | 757 | 520 | 705 |
| log10 MSD:  Regular mucus | 451 | 643 | 308 | 673 |
| log10 MSD:  MUC5AC KO | 329 | 514 | 359 | 945 |
| Pore size:  MUC5B KO | 197 | 377 | 248 | 358 |
| Pore size:  Regular mucus | 225 | 344 | 162 | 356 |
| Pore size:  MUC5AC KO | 211 | 297 | 208 | 506 |
| Microviscocity:  MUC5B KO | 353 | 756 | 524 | 717 |
| Microviscocity:  Regular mucus | 447 | 639 | 312 | 686 |
| Microviscocity:  MUC5AC KO | 328 | 506 | 366 | 958 |
